# Degradation Investigation of Selected Taste and Odor Compounds by a UV/Chlorine Advanced Oxidation Process

**DOI:** 10.3390/ijerph15020284

**Published:** 2018-02-07

**Authors:** Jingyun Fang, Jiajian Liu, Chii Shang, Chihhao Fan

**Affiliations:** 1 Guangdong Provincial Key Laboratory of Environmental Pollution Control and Remediation Technology, School of Environmental Science and Engineering, Sun Yat-Sen University, Guangzhou 510275, China; fangjy3@mail.sysu.edu.cn; 2 Department of Civil and Environmental Engineering, The Hong Kong University of Science and Technology, Clear Water Bay, Kowloon, Hong Kong, China; jliubj@connect.ust.hk (J.L.); cechii@ust.hk (C.S.); 3 Department of Bioenvironmental Systems Engineering, National Taiwan University, Taipei 10617, Taiwan

**Keywords:** taste and odor compounds, UV/chlorine advanced oxidation process, hydroxyl radical, chlorine radical, water treatment

## Abstract

Taste- and odor-causing (T&O) compounds are a major concern in drinking water treatment plants due to their negative impacts on the safety and palatability of water supply. This study explored the degradation kinetics and radical chemistry of four often-detected T&O compounds, geosmin (GSM), 2-methylisoborneol (MIB), benzothiazole (BT), and 2-isobutyl-3-methoxypyrazine (IBMP), in the ultraviolet/chlorine (UV/chlorine) advanced oxidation process. All experiments were carried out in a 700 mL photoreactor and the process effectively degraded the investigated T&O compounds in a slightly acidic environment. The degradation of T&O decreased with increasing pH but slightly with decreasing chlorine dosage. When the pH increased from 6 to 8, the pseudo-first-order rate constants of GSM, MIB, BT, and IBMP dropped from 2.84 × 10^−3^, 2.29 × 10^−3^, 3.64 × 10^−3^, and 2.76 × 10^−3^ s^−1^ to 3.77 × 10^−4^, 2.64 × 10^−4^, 6.48 × 10^−4^, and 6.40 × 10^−4^ s^−1^, respectively. Increasing the chlorine dosage slightly accelerated the degradation of the investigated T&O compounds, but excessive hypochlorous acid and hypochlorite scavenged the HO• radicals and reactive chlorine species (RCS). Generally, HO• primarily contributed to the degradation of all of the investigated T&O compounds as compared to RCS. The degradation by RCS was found to be structurally selective. RCS could not degrade GSM, but contributed to the degradation of MIB, BT, and IBMP. The results confirmed that the proposed oxidation process effectively degraded typical T&O compounds in aqueous phase.

## 1. Introduction

The treatment of taste and odor (T&O) compounds has become a priority task for drinking water supply. Although the aqueous concentrations of T&O compounds are usually at a relatively low range, around ng/L, their occurrence still attracts questions and complaints regarding the issue of the safety and palatability of drinking water. Often-found T&O compounds in natural waterbodies include geosmin (GSM), 2-methylisoborneol (MIB), 2-isobutyl-3-methoxypyrazine (IBMP), and benzothiazole (BT). The odor thresholds of GSM, MIB, IBMP, and BT are 4, 15, 1, and 80 ng/L, respectively [1], while the reported levels of these compounds in surface waters range from 10 to 1000 ng/L [2,3,4], leaving a difficult task for drinking water treatment processes.

Both GSM and MIB contain tertiary alcohol groups, which make them difficult to oxidize, and their low volatility also impedes their removal by air stripping. In addition, conventional treatment processes, such as coagulation, precipitation, sand filtration, and oxidation, are inefficient at removing these compounds [5,6]. Membrane separation can effectively remove GSM and MIB from water at a relatively high operational expense, but the issue of surficial fouling is another major concern [7,8]. Using a biological process alone to remove GSM and MIB is not as effective, since the biodegradation rates of GSM and MIB are slow [9,10]. Although activated carbon is widely used in removing GSM, MIB, and IBMP, the co-existing natural organic matters in waterbodies can reduce its adsorption capacity for T&O compounds as well as the treatment efficiency [11,12,13,14]. BT has a relatively high aqueous solubility and polarity due to its low octanol–water partition coefficient (i.e., log K_OW_ = 1.99–2.41) [15], suggesting that BT is difficult to sorb onto particles, settle to sediments, or be bioaccumulated. BT can be microbially degraded but its transformation rate is relatively low [16].

Advanced oxidation processes (AOPs) are attractive technologies to remove T&O compounds by generating reactive radicals, including the hydroxyl radical (HO•, E_0_ = 2.8 V) or sulfate radicals (SO_4_^•−^, E_0_ = 2.3–2.8 V). The ultraviolet/hydrogen peroxide (UV/H_2_O_2_) system has been widely used in drinking water treatment, and this process also exhibits an excellent removal efficiency for T&O compounds, such as GSM, MIB, and BT [1,3,17]. However, major disadvantages of the UV/H_2_O_2_ process include (1) incurred costs associated with the chemical addition of H_2_O_2_ and its residual quenching agents and (2) the operational challenge of balancing peroxide quenching with the needs of secondary disinfection [18].

Recently, the UV/chlorine process has been considered as an emerging alternative to the UV/H_2_O_2_ process, as it is energy-efficient and capable of removing a variety of recalcitrant contaminants. The UV/chlorine process was reported to be effective in degrading T&O compounds of GSM and MIB, and other contaminants, such as pharmaceutical and personal care products (PPCPs), trichloroethylene, and benzoic acids [18,19,20,21,22,23,24]. The mechanism of the UV/chlorine process is shown in Equations (1)–(3). When aqueous free chlorine is exposed to UV, HO• and Cl• are generated [22]. Meanwhile, Cl• can react with Cl^−^ to form Cl_2_•^−^ (Equation (4)). Consequently, in the UV/chlorine system, HO•, Cl•, and Cl_2_•^−^ are the main radicals that react with contaminants. Furthermore, Cl• is rather selective, in that it has strong reactivity with electron-rich organics, such as phenol, dimethylaniline, and toluene, but less reactivity with electron-poor organics, such as nitrobenzene (NB) [25,26,27].

HOCl + hv → HO• + Cl•(1)

OCl^−^ + hv → O•^−^ + Cl• (2)

O•^−^ + H_2_O → HO• + OH^−^ (3)

Cl• + Cl^−^ ⇆ Cl_2_•^−^ (4)

The coexistence of various reactive species of HO•, Cl•, and Cl_2_•^−^ in the UV/chlorine system makes it different from other AOPs that rely on HO• only (i.e., UV/H_2_O_2_). Several studies investigated the effectiveness of the UV/chlorine system in removing GSM and MIB [4,18,19]. Kim et al. (2016) studied the removal of GSM and MIB under a UV fluence of 90,000 J m^−2^. The degradation was more efficient in the acidic environment [4]. However, the radical chemistry of the degradation of the two investigated T&O compounds remains unclear. In the study by Watts et al. (2012), HO• was considered as the only oxidant for T&O degradation [18]. The degradation resulting from other reactive species in the UV/chlorine system is not well-studied. Whether the attacks by the reactive chlorine species (RCS) would accelerate the degradation kinetic of T&O compounds deserves further investigation.

According to the aforementioned assertion, this study aimed to investigate the efficiency of the UV/chlorine system in degrading the T&O compounds, the influence of various reaction conditions, and the contribution of involving radicals in the T&O degradation. Four typical aqueous T&O compounds, i.e., GSM, MIB, BT, and IBMP (shown in Table 1), were selected as the target compounds because their existence in surface waterbodies has often been reported.

## 2. Materials and Methods

### 2.1. Reagents and Materials

All of the solutions were prepared with ultrapure water (Milli-Q ultrapure water system), stored in a 4 °C thermostatic chamber in the dark, and restored at room temperature before use. GSM and MIB (purity ≥ 98%) were obtained from Wako (Tokyo, Japan); BT and IBMP (purity ≥ 99%) were obtained from J&K (Beijing, China). The concentration of the stock solution was 100 mg/L. Sodium hypochlorite and ammonium chloride were analytically pure. A buffer solution composed of disodium and monopotassium phosphates was used in the present study.

### 2.2. Analytical Methods

GSM and MIB were analyzed by gas chromatography-mass spectrometry (Agilent Technologies 7080A GC-5977A MSD, Santa Clara, CA, USA) using the HP-5MS chromatography column (30 m × 0.25 mm × 0.25 μm), with a flow rate of 1 mL/min and an injection volume of 1 μL. For selected ion monitoring, the corresponding mass/charge ratios were 112 and 95 for GSM and MIB, respectively. BT and IBMP were analyzed by high-performance liquid chromatography using the Poroshell 120 EC-C18 chromatography column (4.6 × 50 mm, 2.7 μm). The flow rate was 1 mL/min, and an injection volume of 50 μL was employed.

The concentration of free chlorine was determined by the DPD colorimetric method and the DPD/FAS titration method [28]. The UV photon flux was measured by the iodide/iodate method [29,30]. The optical path length was determined by measuring the photolysis kinetics of H_2_O_2_ [31,32].

### 2.3. Experimental Procedures

A low-pressure (LP) UV lamp (Heraeus, GPH 212T5L/4, 10W, 254 nm, Hanau, Germany) in a quartz sleeve was placed in the centerline of a 700 mL cylindrical glass reactor with rapid mixing provided at the bottom of the reactor. A water-circulating system kept the inner tank temperature at 25 ± 0.1 °C.

All experiments were carried out in the 700 mL photoreactor described above. The UV lamp and the water-circulating system were heated for at least 30 min before experiments. Experiments were initiated by adding the NaClO stock solution to the UV reactor containing the solution spiked with T&O compounds (GSM and MIB at 400 nM; BT and IBMP at 1 μM) and 2 mM phosphate buffer to maintain an initial chlorine concentration of 5, 20, or 50 μM and pH of 6, 7, or 8. Because of the volatility of the investigated T&O compounds, the device was sealed airtight throughout the reaction process. In the GSM and MIB experiments, 4 mL of the sample was collected at different time intervals then extracted with 3 mL of hexane. After standing for 30 min, 1 mL of the supernatant was taken for GC-MS analysis. In the BT and IBMP experiments, 1 mL of sample was collected for HPLC analysis. In all of the experiments, suitable amounts of ammonium chloride solution (at a molar ratio of ammonium chloride/chlorine of 1.5) were added to quench the reactions before further extraction or analysis. Control tests of T&O compounds degradation by UV direct photolysis and dark chlorination were also conducted in a similar manner but in the absence of chlorine and UV light, respectively.

## 3. Results and Discussion

### 3.1. Degradation Kinetics of T&O Compounds by the UV/Chlorine Process

**Figure 1:**
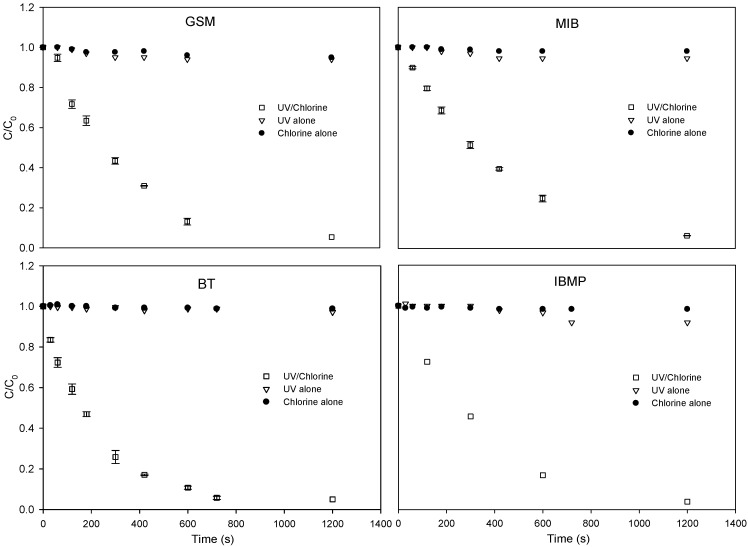
Time-dependent degradation of GSM, MIB, BT, and IBMP in pure water through oxidation by UV/chlorine, UV alone, and chlorination alone. Conditions: pH = 6; chlorine concentration = 50 μM; GSM and MIB concentration = 400 nM; BT and IBMP concentration = 1 μM; nitrobenzene (NB) concentration = T&O compound concentration; Incident photon flux = 0.69 μEinstein L^−1^ s^−1^.

Figure 1 presents the relative concentration changes of GSM, MIB, BT, and IBMP with respect to time for the three investigated methods (compounds by UV/chlorine process, direct UV photolysis, and direct chlorination). The direct UV photo-degradation or direct chlorination approach slightly decreased the concentrations of the four investigated T&O compounds. Contrarily, the UV/chlorine process was more effective than UV photolysis or chlorination alone in removing all four compounds. In the UV/chlorine system, the pseudo-first-order degradation was assumed, and the rate constants of degrading GSM, MIB, BT, and IBMP within 20 min were calculated to be 2.84 × 10^−3^, 2.29 × 10^−3^, 3.64 × 10^−3^, and 2.76 × 10^−3^ s^−1^, respectively. The differences among the UV photolysis, chlorination alone, and UV/chlorine processes can be attributed to the generation of highly oxidative free radicals (HO• and RCS). Note that these obtained rate constants are significant in a relative sense, since they are proportional to the incident photon flux as well as other experimental parameters.

Under illumination by the low-pressure UV lamp (λ = 254 nm), the quantum yields of HO• generated by hypochlorous acid (HOCl) and hypochlorite (OCl^−^) were 1.45 molE s^−1^ and 0.97 molE s^−1^, respectively. The respective molar extinction coefficients were 59 and 66 M^−1^ cm^−1^ [26]. According to the study by Jin et al. [22], the HOCl and OCl^−^ have the quantum yields comparable to that of H_2_O_2_, but the molar extinction coefficients of HOCl and OCl^−^ are higher. Therefore, the UV/chlorine process generated a significant amount of HO• that makes the system a very effective one for organic contaminant removal as compared to the UV/H_2_O_2_ system. However, free radicals could also be scavenged by HOCl and OCl^−^.

#### 3.1.1. Influence of pH

**Figure 2:**
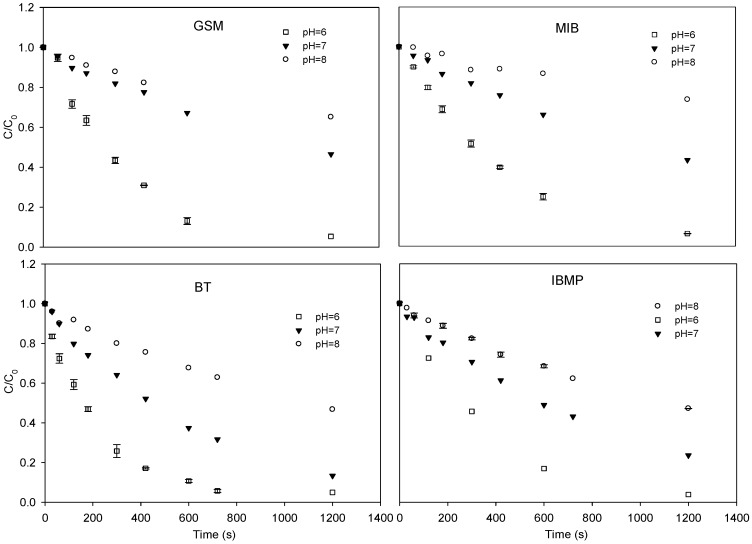
Time-dependent degradation of GSM, MIB, BT, and IBMP in pure water through the UV/chlorine process at different pH levels. Conditions: pH = 6, 7, and 8; chlorine concentration = 50 μM; GSM and MIB concentration = 400 nM; BT and IBMP concentration = 1 μM; NB concentration = T&O compound concentration; Incident photon flux = 0.69 μEinstein L^−1^ s^−1^.

Figure 2 presents the kinetic degradation of GSM, MIB, BT, and IBMP in the UV/chlorine process under various pH conditions. The four T&O compounds demonstrated similar degradation trends under the tested pH conditions. The degradation rate decreased as the pH increased. As the pH was increased from 6 to 8, the pseudo-first-order rate constants of GSM, MIB, BT, and IBMP decreased from 2.84 × 10^−3^, 2.29 × 10^−3^, 3.64 × 10^−3^, and 2.76 × 10^−3^ s^−1^ to 3.77 × 10^−4^, 2.64 × 10^−4^, 6.48 × 10^−4^, and 6.40 × 10^−4^ s^−1^, respectively.

The pH dominates the dissociation of HOCl/OCl^−^ (pKa = 7.5, 25 °C), and the impact of such dissociation on the degradation of T&O compounds can be explained from two aspects. Firstly, pH influenced the quantum yield, which in turn controlled the generation of HO• and Cl•. At room temperature with UV exposure at a 254 nm wavelength, the quantum yield of HOCl was higher than that of OCl^−^. As the pH increased, the quantum yield as well as HO• generation increased, and Cl• concentration decreased. Secondly, pH also influenced the rate of HO• consumption by HOCl and OCl^−^, as indicated in Equations (5)–(8) [26]. When the pH was above 7.5, under which circumstance OCl^−^ became the dominant species in the solution, the consumption rates of the two free radicals were substantially higher than those when the pH was below 7.5.

HO• + HOCl → H_2_O + ClO•  k = 2.0 × 10^9^ M^−1^ s^−1^ (5)

HO• + OCl^−^ → OH^−^ + ClO•   k = 8.8 × 10^9^ M^−1^ s^−1^ (6)

Cl• + HOCl → H^+^ + Cl^−^ + ClO• k = 3.0 × 10^9^ M^−1^ s^−1^ (7)

Cl• + OCl^−^ → Cl^−^ + ClO•    k = 8.2 × 10^9^ M^−1^ s^−1^ (8)

#### 3.1.2. Influence of Chlorine Concentration

**Figure 3:**
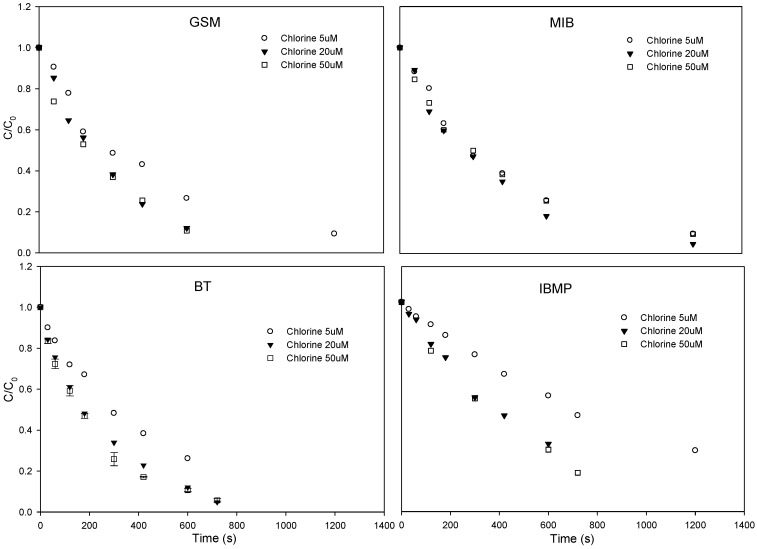
Degradation of GSM, MIB, BT, and IBMP in pure water through the UV/chlorine process at different chlorine concentrations. Conditions: pH = 6; chlorine concentration = 5, 20, and 50 μM; GSM and MIB concentration = 400 nM; BT and IBMP concentration = 1 μM; UV intensity = 0.69 μEinstein L^−1^ s^−1^.

The degradation of the investigated T&O compounds with respect to time at various chlorine concentrations is shown in Figure 3. Although increasing the initial chlorine concentration enhanced the degradation of the T&O compounds, the rate of degradation enhancement gradually declined as the chlorine concentration continued to increase. As the chlorine concentration was increased from 5 to 20 μM, the pseudo-first-order rate constant of GSM, MIB, BT, and IBMP increased from 2.05 × 10^−3^, 2.06 × 10^−3^, 2.29 × 10^−3^, and 1.42 × 10^−3^ s^−1^ to 3.43 × 10^−3^, 2.62 × 10^−3^, 3.85 × 10^−3^, and 2.55 × 10^−3^ s^−1^, respectively. When the chlorine concentration was further increased to 50 μM, the pseudo-first-order rate constant of GSM, BT, and IBMP increased to 3.53 × 10^−3^, 4.00 × 10^−3^, and 3.76 × 10^−3^ s^−1^, respectively; while MIB decreased to 2.08 × 10^−3^ s^−1^.

As shown in Equations (5)−(8), the free chlorine consumes the HO• and Cl•, and all of them have the reaction rate at the order of 10^9^ M^−1^ s^−1^ (2.0 × 10^9^ to 8.8 × 10^9^ M^−1^ s^−1^). These reaction rates are comparable to those of the selected target T&O compounds with HO• (5.10 × 10^9^ to 1.18 × 10^10^ M^−1^ s^−1^), implying that an overdose in free chlorine may facilitate the competition with the T&O compounds for the HO• in the system, causing rapid consumption of HO• and therefore the decline in the T&O compound degradation.

### 3.2. Roles of OH• and RCS

In the contribution differentiation of target T&O compound degradation by different radicals, NB was selected as reference material because, in the UV/chlorine system, it reacts with HO• in a markedly fast fashion (at a second-order rate constant of 3.9 × 10^9^ M^−1^ s^−1^) as compared to the reaction with RCS, which is considered negligibly slow. By assuming that the concentration of hydroxyl radical remained invariant during the degradation, the degradation process can be viewed as a pseudo-first-order decay with respect to the target compound. Therefore, through a competition kinetic analysis, the steady-state concentrations of HO• in the UV/chlorine system under the tested pH conditions can be obtained (shown in Table 2). The steady-state concentration represents the overall HO• balance after considering its generation and consumption in the system. By multiplying the steady-state concentration of HO• and the second-order rate constants of the investigated T&O compounds (shown in Table 1), the observed rate constants between HO• and the investigated T&O compounds can be calculated, and the contributions of HO• and RCS to the degradation of target substances can be further explored. However, it can be observed from Figure 2 that the degradation becomes more apparent as the pH decreased, and this HO• concentration variation exhibits a similar trend (as shown in Table 2). This also implied that OH• may be the most important oxidant in the system that is responsible for target compound degradation, and further discussion is introduced in the following section.

**Figure 4:**
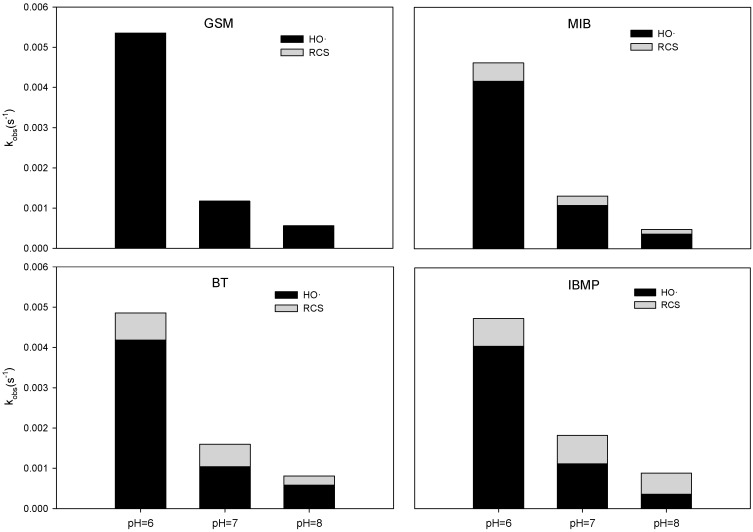
Pseudo-first-order rate constants (k_obs_) of the degradation of T&O compounds by HO• and reactive chlorine species (RCS) (e.g., Cl• and Cl_2_•^−^) in the UV/chlorine process at different pH values. Conditions: pH = 6, 7, and 8; chlorine concentration = 50 μM; GSM and MIB concentration = 400 nM; BT and IBMP concentration = 1 μM; NB concentration = T&O compound concentration; UV intensity = 0.69 μEinstein L^−1^ s^−1^.

Figure 4 shows the contributions of various free radicals to the degradation of GSM, MIB, BT, and IBMP. As the pH increased, the pseudo-first-order rate constants of GSM, MIB, BT, and IBMP in the UV/chlorine system decreased gradually. For GSM at pH 6, 7, and 8, the contribution of HO• was almost 100%, since contributions from RCS were negligible. For MIB at pH 6, the contribution of HO• was 90%, and at pH 8, it decreased to 77%, while the contribution of RCS increased to 23%. These results may be because both GSM and MIB are of a tertiary alcohol and lack an electron-donating group in their structure. It is difficult for RCS, such as Cl•, to induce an oxidation or substitution reaction. Similar phenomena were observed for BT and IBMP. For BT, at pH 6, the contributions of HO• and RCS were 86% and 14%, respectively; at pH 8, the corresponding contributions were 72% and 28%, respectively. For IBMP, at pH 6, the contributions of HO• and RCS were 85% and 15%, respectively; at pH 8, the corresponding contributions were 40% and 60%, respectively.

## 4. Conclusions

The UV/chlorine process effectively degraded the four investigated T&O compounds (GSM, MIB, BT, and IBMP). As the pH increased from 6 to 8, the pseudo-first-order rate constants of GSM, MIB, BT, and IBMP decreased from 2.84 × 10^−3^, 2.29 × 10^−3^, 3.64 × 10^−3^, and 2.76 × 10^−3^ s^−1^ to 3.77 × 10^−4^, 2.64 × 10^−4^, 6.48 × 10^−4^, and 6.40 × 10^−4^ s^−1^, respectively. The increase in chlorine concentration accelerated the degradation of the T&O compounds, but excessive HOCl and OCl^−^ removed the HO• from the system. Kinetic analyses of the competitive reactions showed that in the UV/chlorine process, the hydroxyl radical primarily contributes to the degradation of the T&O compounds as compared to RCS under most of the pH levels tested. Nonetheless, the degradation by RCS was structurally specific to the target compound. RCS could not degrade GSM, but contributed to the degradation of MIB, BT, and IBMP, particularly at alkaline pH. The results of this study verify that the UV/chlorine process can effectively degrade typical T&O compounds in an aquatic environment.

## Figures and Tables

**Table 1:** Properties of selected taste and odor (T&O) compounds in this study.

Name/CAS Number	Formula	Molecular Weight (g/mol)	Odor Threshold Value (ng/L)	K_OW_/K_h_ (Pa m^3^ mol^−^^1^)	k_HO•_ (×10^9^ M^−^^1^ s^−^^1^)
GSM/16423-19-1	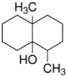	182.30	10	3.70/10^−5^	11.78 * 8.20~14.0 [16,17]
MIB/2371-42-8	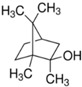	168.28	10~15	3.13/10^−5^	6.26 * 5.10~8.10 [16,17]
BT/95-16-9	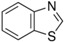	135.19	80	1.99–2.41	9.92 * 8.61 [3]
IBMP/24683-00-9	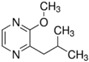	166.22	1		7.17 *

Note: GSM: geosmin; MIB: 2-methylisoborneol; BT: benzothiazole; IBMP: 2-isobutyl-3-methoxypyrazine. Kow = octanol–water partition coefficient; Kh = Henry’s law constant; k_HO•_ = second-order rate constant with HO•; * = data obtained by this study.

**Table 2:** Steady-state concentration of HO• in the UV/chlorine advanced oxidation processes (AOPs). Condition: pH = 6; chlorine dosage = 50 μM; GSM and MIB dosage = 400 nM; BT and IBMP dosage = 1 μM; NB concentration = T&O compound concentration; UV intensity = 0.69 μEinstein L^−1^ s^−1^.

T&O	HO• Concentration (×10^−14^ M)		
	pH 6	pH 7	pH 8
GSM	34.1	7.50	3.58
MIB	39.1	10.1	3.44
BT	35.6	5.78	4.22
IBMP	30.4	8.43	2.69
